# Comparing the Effect of Oral Supplementation of Vitamin E, Injective Vitamin E and Selenium or Both during Late Pregnancy on Production and Reproductive Performance and Immune Function of Dairy Cows and Calves

**DOI:** 10.1155/2014/165841

**Published:** 2014-06-18

**Authors:** Farokh Kafilzadeh, Habibollah Kheirmanesh, Hamed Karami Shabankareh, Mohhamad Reza Targhibi, Elaheh Maleki, Mahdi Ebrahimi, Goh Yong Meng

**Affiliations:** ^1^Department of Animal Science, Faculty of Agriculture, Razi University, Kermanshah, Iran; ^2^Department of Veterinary Preclinical Sciences, Faculty of Veterinary Medicine, Universiti Putra Malaysia (UPM), 43400 Serdang, Selangor, Malaysia; ^3^Institute of Tropical Agriculture, Universiti Putra Malaysia, 43400 Serdang, Selangor, Malaysia

## Abstract

The object of this study was to determine the effect of prepartum supplementation of vitamin E with or without injective vitamin E and selenium (Se) on productive and reproductive performances and immune function in dairy cows. Sixty multiparous Holstein dairy cows were divided randomly into three groups at the end of gestation. Cows in each group received one of three treatments: (1) a single intramuscular (im) injection of vit. E + selenium 3 weeks prepartum; (2) daily supplementation of oral vit. E given from 3 weeks prepartum to parturition; (3) injective vit. E + Se with daily supplementation of oral vit. E. Blood samples were collected from cows at calving and from calves at 0 and 7 days of age. Concentration of IgG in serum of cows and calves as well as in colostrum was determined. No significant differences among treatments occurred in the concentrations of IgG, animal, and calf production and reproduction performance. Due to the lack of significant difference between injection and oral supplementation, it is recommended to replace the injection with oral supplementation.

## 1. Introduction 

Most diseases in dairy cows occur at or just after calving, which is a period associated with immune suppression, resulting in an increased susceptibility to infections [1, 2]. Prepartum immune suppression is multifactorial but is associated with endocrine changes and decreased intake of critical nutrients [3]. Vitamin E is a fat-soluble vitamin and is not synthesized in the rumen. The vitamin E requirement must therefore be provided in the field. However, the vitamin E content of the basal diet is highly variable and is not known in most situations. Although vitamin E content is high in fresh grass, it markedly reduces during storage and conservation [4]. Therefore, NRC [5] recommends that the total vitamin E requirement should be given via dietary supplements when conserved forages are fed and that extra supplementation may be useful during periods of immune suppression, such as around calving. Vitamin E and Se are essential micronutrients that share a common biological role as antioxidants [6, 7]. The vitamin E (*α*-tocopherol) status of dairy cows is one important component of a well-functioning immune system because of its antioxidant effects on cows [8–10] and young dairy calves [11, 12]. At parturition, plasma concentrations of vitamin E were found to decrease by 47% [13], because of secretion of the vitamin into the udder during colostrogenesis, decreased dry matter intake (DMI) at calving, and an increased need for antioxidants during this time [13–15]. Passive transfer of colostral immunoglobulins is vital to short-term health and survival of neonates, and limited data suggest that inadequate transfer occurs in 10 to 25% of newborn beef calves [16]. Low serum immunoglobulins in young calves were related to increased incidence of disease [17]. Previous investigations have revealed that serum IgG concentrations decreased at parturition [18, 19].

Antioxidants are necessary to prevent some disorders in female reproduction [20]. Effects of Se, vitamin E, or their combination on fertility have been variable, with some studies reporting no effect [21] or an increase in fertility [22, 23]. Retained fetal membranes (RFM) occur when the placenta has not been shed within a short time after parturition. Cows with RFM have increased risks for metritis [24, 25] and mastitis [26] during early lactation. Some researchers [27] have observed a reduction in the incidence of RFM with a single injection of Se and vitamin E given approximately 21 d prepartum. In contrast, other researchers [23, 28, 29] have found no benefit of vitamin E or Se.

Smith et al. [30] were the first to report a beneficial effect of vit. E supplementation on incidence of mastitis. Feeding vit. E during dry periods until 30 days after calving resulted in 80% decrease in clinical mastitis and 60% reduction in intramammary infections [31] while Batra et al. [32] found no benefit of vit. E supplementation.

Lacetera et al. [33] reported that milk yield was higher in cows treated with Se and vitamin E than nontreated cows, but Weiss and Spears [10] showed no beneficial effect of Se or vitamin E supplementation on milk production.

Effects of Se and/or vitamin E on calf weight have been variable, with some studies reporting benefit effect [34, 35] and some reporting no effect [36, 37].

It is a common practice in all dairy farms in Iran to give an injective dose of vitamin E and Se 21 days prior to calving. Previous study [37] from our colleagues indicated a beneficial effect of double injection of vitamin E and Se (each mL contained 0.5 mg Se as sodium selenite and 50 IU of d-l-*α*-tocopheryl acetate) over a single injection 21 days prepartum. However, it was not clear whether that was due to the extra vitamin E, selenium, or both. The objectives of this study were to determine the effect of prepartum dietary supplementation of vitamin E in addition to or without injective vitamin E + selenium during late pregnancy on production and reproduction performance and immune system of multiparous cows and their calves.

## 2. Materials and Methods

This study was conducted in a large commercial dairy farm in Kermanshah province in the west of Iran. The research protocol was approved by Razi University Animal Care and Use Committee. Sixty multiparous Holstein dairy cows in late gestation were randomly assigned into three groups. Cows in each group received one of three treatments: (1) a single intramuscular injection of 20 mL vit. E + selenium, 3 weeks prepartum (each mL contained 0.5 mg Se as sodium selenite and 50 IU of d-l-*α*-tocopheryl acetate); (2) oral supplementation of vit. E (4000 IU vit. E from d-l-*α*-tocopherol acetate) given from 3 weeks prepartum to parturition; (3) injective vit. E + Se plus oral vit. E. Animals were fed a total mixed ration (TMR) containing alfalfa hay, corn silage, and concentrate according to NRC [5] (Table 1).

The calves were separated immediately after birth (within 15 min) and received 2 L of colostrum followed by feeding another 2 L in 8–10 hours by a nursing bottle tube. Blood samples were collected from cows at calving and from calves at 0 and 7 days of age. All blood samples were centrifuged for serum collection and then stored at −20°C. Concentrations of IgG in serum and colostrum were determined by sandwich ELISA.

After parturition, the animals were moved in with the lactating herd. Monthly milk productions of animals were recorded every 15 days until 90 DIM. Calf birth weight, the mean daily gain, and weaning weights, calving to placenta expulsion, were recorded. The case definition for RFM was failure to pass the fetal membranes by 12 h after calving and for clinical mastitis was a producer diagnosis of abnormal milk or swelling of the udder, including cows with systemic illness attributed to mastitis, within 3 months after calving.

Duncan's multiple range tests were used to test differences between means once a significant effect of treatment was indicated by ANOVA. All statistical analyses were performed using SPSS package 16 [38]. The model used is described as follows:

(1) Xij=μ+Tj+εij,

where *X*_*ij*_ is dependent variable, *μ* is the overall mean, *T*_*j*_ is the effect of treatment, and *ε*_*ij*_ is the random error.

## 3. Results and Discussion

No significant differences among treatments occurred in the concentrations of IgG in serum and colostrum of dairy cows (Table 2). Reddy et al. [39] observed a trend for greater titer values for those given 125 IU of vitamin E daily compared with cattle receiving no vitamin E. But Lacetera et al. [33] found that administration of 5 mg of Se in sodium selenite form and 25 IU of vitamin E/100 kg of body weight of cows did not affect plasma IgG concentrations in cows. Similarly, no change in IgG was observed when ewes were supplemented with vitamin E [40].

Because of placenta layer in cattle, there was no placental transfer of immunoglobulin, and the newborn calf is dependent upon colostrum for passive immunity [39]. The lack of difference in serum IgG of calves, in the present study, supplemented with both forms of vit. E could probably be due to the variation in absorptive ability among calves or to the sufficiency of vit. E used in only injective or oral supplemented groups, which could have partially masked the enhancing effect. A previous report from the same farm showed no significant differences in concentration of IgG in serum and colostrum. No change in colostral IgG to vit. E supplementation has been reported by others in ewes [40] and in cows [33, 41].

Effect of treatment on birth weight, daily gain, and weaning weight was not significant because of the sufficiency of vitamin E and Se level in all experimental groups (Table 3). Cohen et al. [42] reported that precalving Se and vitamin E injections had no effect on calf birth weight, daily gain, and weaning weight compared with control, when cows were in marginal (95 *μ*g/L) selenium status. Other investigators have also reported no significant impact of Se and/or vit. E supplementation of dams on body weight gain of their calves [21, 36, 37].

There was no effect of treatment on milk yield, fat percentage and yield, and the incidence of clinical mastitis (Tables 4 and 5). It is generally accepted that the performance of lactating dairy cows can be affected by vitamin E and Se status during the parturient period [33, 43]. Lacetera et al. [33] reported that milk production increased by 10% in cows supplemented with 25 IU of vitamin E and 5 mg Se per 100 kg of body weight. Moeini et al. [37] also showed that milk production during 8 weeks of lactation was higher when a double injection of vitamin E and Se (each injection contained 1000 IU vitamin E and 10 mg Se) was given, but this effect was not observed when milk yield during 12 weeks was compared between cows that received a single or double dose or injective vitamin E and Se. Weiss and Spears [10] and Bourne et al. [44] found no significant effect on milk production due to vit. E supplementation.

Days open and services per conception did not differ between experimental groups (Table 5). Reports on the effects of Se, vitamin E, or their combination with fertility are variable, with some studies reporting an increase in fertility [2, 22, 34] and some reporting no effect [37, 44].

The reasons of these discrepancies could be related to the level of Se, interaction of Se-vit. E, and other nutrition factors as protein, energy, Ca, Mg, and P intake that might also influence reproduction rates [45]. Differences between studies in the amount of vit. E and/or Se administered, the period of administration, and nutritional status of the experimental animals with respect to vit. E and Se intake could explain some of these differential results [2].

## 4. Conclusions

Due to the lack of significant difference between treatments and ease of use of oral supplements, oral supplementation is recommended to replace the injection. No advantage of oral supplementation plus injective vitamin E compared to either of them alone could be due to the sufficiency of vitamin E in all treatments. As discussed above, the illnesses suffered after birth were suitable indicators showing good nutritional and other management conditions under which the dairy herd was kept.

## Figures and Tables

**Table 1:** Nutrient composition of precalving and postcalving experimental diets.

Item	Precalving	Postcalving
NE_l_ (Mcal/Kg DM)	1.52	1.69
CP (%)	15.3	19.3
RDP (%)	10.5	12.1
NDF (%)	41.8	33.9
NFC (%)	36	39.5
Ca (%)	0.71	0.95
P (%)	0.32	0.4

NE_l_: net energy for lactation; CP: crude protein, RDP: ruminant degradable protein; NDF: neutral detergent fiber, NFC: nonfiber carbohydrate.

**Table 2:** Effect of oral supplementation of vitamin E with or without injective vitamin E on immunoglobin concentrations (mg/dL) in blood and colostrums.

Immunoglobulin concentration (mg/dL)		Treatments	
	Injection	Injections + oral	Oral
Cattles blood serum at parturition	2039 ± 39	2128 ± 40	2118 ± 38
Calves blood serum at birth	132 ± 17	153 ± 22	129 ± 22
Calves blood serum at day 7	1312 ± 27	1279 ± 22	1216 ± 22
Colostrum at parturition	6097 ± 32	6012 ± 31	6346 ± 34

**Table 3:** Effect of oral supplementation of vitamin E with or without injective vitamin E on body weight of calves.

Item	Treatments		
	Injection	Injections + oral	Oral
Birth weight	43.35 ± 3.71	42.55 ± 4.34	42.86 ± 4.08
Weaning weight	94.18 ± 9.19	89.56 ± 12.81	97.35 ± 9.84
Body weight gain	50.82 ± 9.36	50.18 ± 14.73	53.30 ± 11.7
Daily body weight gain	0.564 ± 0.10	0.557 ± 0.16	0.592 ± 0.13

**Table 4:** Effect of oral supplementation of vitamin E with or without injective vitamin E on milk yield in multiparous dairy cows.

Item	Treatments		
	Injection	Injections + oral	Oral
Milk production			
First month	31.44 ± 9.31	35.33 ± 8.83	36.63 ± 7.59
Second month	32.16 ± 9.68	34.53 ± 8.90	34.79 ± 7.06
Third month	31.29 ± 7.74	30.07 ± 6.24	33.64 ± 7.63
Fat (percentage)			
First month	2.728 ± 0.64	2.37 ± 0.57	2.483 ± 0.55
Second month	2.681 ± 0.10	2.573 ± 0.10	2.501 ± 0.06
Third month	2.721 ± 0.53	2.819 ± 0.43	2.680 ± 0.62
FCM			
First month	24.65 ± 4.93	26.11 ± 4.71	27.81 ± 4.15
Second month	24.91 ± 5.53	26.41 ± 4.36	26.58 ± 3.79
Third month	24.78 ± 4.67	24.40 ± 3.50	26.40 ± 3.97

**Table 5:** Effect of oral supplementation of vitamin E with or without injective vitamin E on reproductive performance in multiparous dairy cows.

Item	Treatments		
	Injection	Injections + oral	Oral
Mastitis	2	1	2
Metritis	2	0	0
Retained placenta	0	0	1
Open days	118.41 ± 46.08	143.06 ± 47.62	118.36 ± 40.90
Service per conception	2.00 ± 1.28	3.07 ± 1.75	2.36 ± 1.21
